# Identifying Participants Who Would Benefit the Most from an Adult Food-literacy Program

**DOI:** 10.3390/ijerph16071272

**Published:** 2019-04-09

**Authors:** Andrea Begley, Ellen Paynter, Lucy M. Butcher, Vanessa Bobongie, Satvinder S. Dhaliwal

**Affiliations:** 1 School of Public Health, Curtin University, Perth 6102, Australia; ellen.paynter@curtin.edu.au (E.P.); s.dhaliwal@curtin.edu.au (S.S.D.); 2 Foodbank Western Australia, Perth Airport 6105, Australia; lucy.butcher@foodbankwa.org.au (L.M.B.); vanessa.bobongie@foodbankwa.org.au (V.B.)

**Keywords:** food literacy, community participation, dietary intake

## Abstract

Food literacy programs aim to improve behaviours required to achieve a quality diet. The objectives of this study were to assess the demographic, food literacy related and dietary behaviour of participants enrolling in Food Sensations^®^ for Adults, a free four-week food literacy program and identify the subgroup of participants who benefit most. Cross-sectional pre-program questionnaire data (n = 1626) from participants enrolling in the program was used to stratify into low, middle and high food-literacy tertiles. Factor scores from a reliability analysis of food literacy behaviours were then used to produce a composite score). Participants were 80.2% female, 56% aged 26 to 45 years and 73.3% from low to middle socio-economic areas. Demographic characteristics were not a significant predictor of the lowest composite food-literacy group. Those with the lowest composite food-literacy tertile score were more likely to have lower self-rated cooking skills, a negative attitude to the cost of healthy foods, lower intakes of fruits and vegetables and a higher frequency of consuming takeaway food and sugary drinks. Food literacy programs must focus on recruiting those who have low self-rated cooking skills, who consider healthy foods expensive and have poor dietary intakes and will most likely to benefit from such programs.

## 1. Introduction

A healthy diet is crucial for a person’s wellbeing and preventing chronic disease [1]. National surveys show that the diets of most Australians are not consistent with the national dietary guidelines [2]. Less than seven per cent of Australians eat the recommended number of serves of vegetables and 74% overconsume energy-dense, nutrient-poor foods [2,3]. Evidence suggests that poor quality diets are, in part, the result of a lack of knowledge and skills to plan, select and prepare healthy meals [4,5]. Food literacy is a term that has emerged to encompass the knowledge, skills and behaviours involved in planning, selecting, preparing and consuming healthy meals and snacks [6,7,8,9,10]. As a result, food literacy is described as a platform to support the development and maintenance of healthy dietary behaviours [6]. The current challenge is to measure the multiple components of food literacy to assess what is required to educate people make to healthy food choices [11,12,13].

Research from developed countries suggest that generally people eat meals prepared at home most days and cooking skills and competence are highly rated [14,15,16]. However, little is known about food literacy behaviours in Australia, as they are not included in monitoring and surveillance efforts in national surveys as in the United Kingdom [15,17,18], the United States [19] and Canada [20]. Market research surveys have shown that, in Australia, general interest in cooking is high. An online survey discovered that two-thirds of adult food preparers wanted to learn more about cooking and that cooking from scratch was practiced [21]. Other market research discovered that, of 1059 randomly selected participants, those with high cooking confidence were more likely to plan meals and report fresh food preparation as of higher importance [22].

From the little data available, high food-literacy behaviours (i.e., confidence and use) is an important predictor of healthy dietary intakes, particularly for fruit and vegetable intake. In a study examining the relationship between confidence to cook, socio-demographic characteristics and the purchase of vegetables for a household in Brisbane, it was found that a greater variety of vegetables were purchased regularly in households in which the main food preparer was confident about their skills and used a variety of cooking techniques [23]. Crawford et al. discovered in a sample of Melbourne women those who planned meals more frequently, wrote a shopping list and who enjoyed shopping and cooking were likely to eat more fruit and vegetables, although the proportion using these food literacy behaviours regularly was small [24].

Food literacy programs, such as cooking skill interventions, are a popular strategy to improve diet quality. However, criticisms of these programs are directed at a perceived selection or participation bias, whereby those attending are more interested in cooking [25,26,27,28]. Those with interest in cooking may be more likely to have high food literacy at the start of a program. Programs generally target vulnerable groups [29], as those with lower incomes are thought to benefit more from improving their confidence and skills. In reality, this socio-economic group may not have the resources to travel to venues to attend the programs [30], the income to change their food purchasing behaviours [31] or the equipment required for healthy food preparation [32,33]. Publications on program effectiveness are limited in their description of the demographic characteristics and reach to enable assessment of participant types [26]. A deeper examination of the characteristics of food literacy program participants will inform how these programs could be targeted in the future.

The objectives of this analysis are to firstly assess the socio-demographic, food literacy related and dietary behaviour characteristics of participants enrolling in an adult food literacy program and secondly to identify the subgroup of participants who will benefit most from food literacy programs.

## 2. Materials and Methods 

### 2.1. Study Design

Foodbank Western Australia (WA) has been delivering an adult food-literacy program to target groups living in low socio-economic circumstances since 1997. They have developed a curriculum suitable for a variety of people [34]. Food Sensations for Adults (FSA) is the current version of the program, which is based on an Australian food-literacy model covering four areas: planning, selection, preparation and eating [6]. Funded by the Western Australian Department of Health for two and half years until June 2018, FSA targeted adults from low to middle incomes with low food literacy who wished to improve their food literacy. The program is promoted as a free four-session nutrition and cooking program in WA. Scheduled programs are open to the general public and the program is also delivered through existing community membership groups in metropolitan and regional areas using face-to-face and video-conferencing delivery methods. Although individuals from low to middle income households are the intended audience, FSA is marketed extensively to all adults via social and traditional media, websites, professional referrals and word of mouth. As a result, the program attracts a diverse range of people. The only inclusion criteria are the ability to shop and cook independently. Childcare is provided for some sessions for parents with children aged up to five years old. Participants attending 223 FSA programs between May 2016 and June 2018 were encouraged to complete a pre-program questionnaire before beginning the first session (n = 2628). Not all programs were evaluated, due to the literacy levels of participants, mental health and disability limitations and not all participants consented to the ethics process. Participants are not reimbursed for completing questionnaires. Data analysis was completed by an independent evaluator with de-identified data to reduce the chance of bias in responses.

### 2.2. Evaluation Tool

The pre-program questionnaire was developed to address the required outcomes of the funder. It included a 14-item food-literacy behaviour checklist, four food-literacy related practices, four short questions about dietary behaviours and eight socio-demographic variables. The development and validation process for the pre-program food-literacy behaviour questionnaire has been previously published [35]. The process considered respondent burden, cognitive load and reading levels of potential participants. The questionnaire was developed by adapting an extensively tested food behaviour checklist used in the Expanded Food and Nutrition Education Program to include 14 food literacy behaviours [36,37,38,39]. Additional questions were selected from the Western Australian Department of Health’s Nutrition Monitoring Surveillance Survey (NMSS) and the latest data from the 2015 survey published in 2017 [40]. These surveys covered the level of responsibility for meal planning and shopping similar to those used in the US National Health and Nutrition Examination Survey [41] and self-rated cooking skills drawn from unpublished qualitative research to inform the Go for 2&5^®^ fruit and vegetable social marketing campaign [42]. An additional practice question was included on attitude to the cost of healthy foods to measure an objective required by the funder. Four short dietary questions were adapted from the same survey series, including two questions about the average consumption of serves of fruits and vegetables and two questions about the frequency of consumption of takeaway foods and sweetened drinks.

The 2015 NMSS was conducted from July to September with a stratified random sample of adults aged 18 to 64 years. Participants were drawn from the 2013 electronic residential telephone listings for WA by area of residence [40]. The survey was conducted using computer-assisted telephone interviews, with a final sample of n = 1207. The data were weighted for sample design and probability of selection. Post-survey adjustments were made to compensate for under or over-representation of sex, age group or area of residence using the 2014 Estimated Resident Population for WA residents aged 18 to 64 years. 

Demographic characteristics collected from participants included sex, age, highest education level, household composition, postcode, birth in Australia and Aboriginal or Torres Strait Islander status. Income as a primary demographic characteristic was extrapolated from a self-reported postcode and converted to the Australian Bureau of Statistic’s Socio-Economic Indexes for Areas (SEIFA) decile ranking of the Index of Relative Socio-economic Disadvantage [43]. Deciles 1 to 4 were considered low income, 5 to 7 were considered middle income and 8 to 10 were considered high income. To identify participant enrolment intentions, a final question asking the reasons for attending was included and participants could indicate more than one reason.

### 2.3. Determining the Low Food-Literacy Behaviour Participant Group

In this paper, an innovative approach to the analysis of questionnaire data was used, in addition to descriptive results. The approach aimed to determine the subgroup of participants considered most at risk of low food literacy, using a quantiles method to determine risk. Quantiles create cut-off points in a distribution of variables with equal probabilities. This is an accepted method used in clinical and epidemiological studies to determine the patient or population subgroups most at risk when the cut-off is unknown. Tertiles of patient and population groups have been determined from variables such as clinical tests, biomedical scores and dietary intakes to derive the lowest tertile group and; therefore, identify the group most at risk of poor outcomes associated with low scores [44,45,46]. This approach was taken to identify those most at risk of low food literacy by determining which participants scored in the lowest tertile for food literacy, and then test associated variables.

Three food literacy behaviour factors—plan and manage, selection and preparation—resulted from an exploratory factor analysis of questions related to areas of food literacy in the pre-program questionnaire that was previously published [35]. Of the 14 food literacy behaviour questions, 11 scored 0.4 and above in factor loadings and were included in one or more of the factors. The following responses were recorded: ‘never’ scored 1, ‘sometimes’ scored 2, ‘most of the time’ scored 3 and ‘always’ scored 4. The response score was multiplied by the factor loading score and summed together to form the food literacy behaviour factor scores [47].

Participants were stratified into low, middle or high food-literacy groups (tertiles) for: a) plan and manage, b) selection and c) preparation, based on the distribution of the scores in the cohort to produce tertile ranges (i.e., three equal groups). This analysis focused on the low tertile group for each of the three factors, which were defined as the lowest scoring third of participants. A composite group was also generated, comprising individuals characterised in the low food-literacy group in one or more of the three components.

### 2.4. Statistical Analysis

Statistical analysis was conducted using SPSS version 25 (IBM, New York, NY, USA). Results were considered statistically significant when *p* < 0.05. Logistic regression analyses were used to determine when variables describing demographic characteristics, food literacy related practices and dietary behaviours contributed to low food-literacy behaviour relating plan and manage, selection and preparation, as well as a food literacy composite group. The variables identified to predict low composite food-literacy behaviour were hypothesised to represent the subgroup of the population most likely to benefit from a program targeting food-literacy behaviours. Univariable regression was used to determine which variables were independent predictors of low food-literacy behaviour. Multivariable logistic regression analyses using forced entry method were undertaken to assess which variables contribute to low food-literacy behaviour. There were 17 variables included in the regression analysis including eight socio-demographic, four food literacy related practices and four dietary behaviours. Most were categorical in presentation (n = 15), in which the odds ratio reflected the likelihood of each category being chosen by the low scoring group, compared with the reference category. Fruit and vegetable intake were entered as a continuous variable; an odds ratio represented the likelihood of remaining in the low scoring group for every serve increase in fruit or vegetables. A score of less than one indicated that they were more likely to be in the middle or high scoring group. The 95% confidence interval shows the range of values around the odds ratio that are believed, with a 95% probability, to contain the true odds ratio value. 

### 2.5. Ethics

Ethics approval was obtained from the Human Research Ethics Committee at Curtin University (RDHS-52-16). At the beginning of the first session, participants were provided with a verbal explanation of the purpose of the research and a written research information sheet. Written consent was obtained prior to questionnaire administration.

## 3. Results

### 3.1. Response Rate

A total of 1626 participants completed the pre-program questionnaire (response rate of 61.8%). Most participants were from metropolitan community groups (60.3%), followed by metropolitan public programs (15.3%) and nearly one-quarter were from regional programs (24.3%). The missing data in the questionnaires were random and no questions were commonly missed.

### 3.2. Demographic Characteristics

FSA participants were mostly female (80.2%), with just over half (56%) aged 26 to 45 years, as seen in Table 1. The most common household composition was a couple with children (35.7%). The majority had qualifications under a bachelor level (75.8%) and were unemployed or not employed for money (66%). Nearly three-quarters (73.3%) of participants were classified as from low or middle incomes, in comparison to 58.5 per cent of the WA population, as measured by the Australian Bureau of Statistics SEIFA [43]. Just over one-half of participants were born in Australia (58.1%) and 7.3% identified as Aboriginal or Torres Strait Islander.

### 3.3. Food Literacy Related Practices and Dietary Behaviours

Participants were more likely to report having the sole responsibility for meals and were less likely to report shared or no responsibility, compared to the NMSS 2015 data (Table 2). They were more likely to have the sole responsibility for shopping and were less likely to have shared or no responsibility for shopping. Compared to the NMSS 2015 data, participants were less likely to report being able to cook almost anything or able to cook a wide variety of foods and were more likely to report being able to cook a meat and vegetable meal, being able to cook to the equivalent of boiling an egg or not being able to cook.

The mean self-reported intake of fruit and vegetables in FSA participants was lower than the amount recommended by the Australian Dietary Guidelines for Adults. The guidelines include two or more serves of fruit and five or more serves of vegetables [48] and are lower than the latest Department of Health WA’s NMSS 2015 data [40]. Nearly one-fifth of WA adults (18.8%) reported drinking sugar-sweetened drinks on the day prior to the NMSS survey. Most WA adults (70%) did not purchase a meal from a food outlet on the day prior to the survey.

The most common reasons for FSA attendance (n = 1619) were to learn about healthy eating (59.1%), followed by gain new cooking ideas (58.8%), prepare healthier meals (50.3%), improve confidence when cooking (42.5%), learn to read food labels (37%), improve food budgeting (36.4%) and prepare children’s lunchboxes and snacks (33.1%).

### 3.4. Variables Predicting Low Food-Literacy Behaviours

Table 3 shows a summary and Table 4 shows the results from the univariable and multivariate analyses of variables related to participant socio-demographic characteristics, dietary behaviours and food literacy related practices. The variables were analysed individually (i.e., univariable) and together (i.e., multivariable) to identify those predicted to have low plan and manage behaviour, low selection behaviour, low preparation behaviour and overall low composite food-literacy behaviour.

Seven predictor variables were associated with the low plan and manage group. These were demographics of age and education, dietary intake of fruit and vegetables, frequency of consumption of takeaway food, in addition to cooking skills and responsibility for shopping. Low scoring participants for plan and manage behaviour were more likely to have finished high school or further training than they were to have only completed some high school. This group were also more likely to be aged between 26 and 45 or 56 and 65 than 18 and 25. Participants with low plan and manage scores were more likely to report poorer cooking skills. They were more than eight times more likely to report that they ‘cannot cook’ or ‘do not cook’ than they ‘can cook almost anything’. They were more than two times more likely to have no responsibility for shopping. They reported higher frequency of takeaway food consumption, were over five times more likely to report eating takeaway food three or more times per week than never. They were likely to report a lower intake of serves of fruits and vegetables.

Six variables were identified as predictors for the low selection group. These participants were more likely to have been born outside Australia. They were two and a half times more likely to report basic cooking skills than being able to ‘cook almost anything’. They were one and a half times more likely to answer ‘not sure’ or ‘agree’ than to disagree that healthy food costs more than unhealthy food. Again, the participants who scored in this group were likely to eat less fruits and vegetables. They were more likely to report consuming sugar-sweetened drinks and were over two times more likely to drink these three or more times a week than never. The low preparation group was associated with six predictors. These participants were more likely to answer ‘not sure’ or ‘agree’ than to disagree that healthy food costs more than unhealthy food. They were twice as likely to have no responsibility for meal choice and preparation, than have all the responsibility. These individuals were over 10 times more likely to report not cooking or basic skills than well-developed cooking skills. They were likely to report a lower intake of servings of fruits and vegetables and were more likely to eat takeaway more than once per week. They were over four times more likely to report eating fast food three or more times per week.

Participants determined as low scoring for one or more of the food literacy behaviour factors were included in the composite factor. No socio-demographic characteristics were associated with this composite group. They were over seven times more likely to report basic skills compared to ‘can cook almost anything’. They were over one and a half times more likely to answer ‘not sure’ or ‘agree’ than to disagree that healthy food costs more than unhealthy food. The composite food-literacy behaviour score was associated with all four dietary intake predictors. They were likely to report a lower intake of fruit and vegetables; for each increase of one serve per day, participants were less than 70 per cent as likely to remain in the low scoring group. They were almost three times more likely to eat takeaway food three or more times per week than never. They were nearly two times more likely to drink soft drink one or more times per week than never.

## 4. Discussion

Little is known about food literacy behaviours and their predictors, leading to assumptions about the best target groups for program investment. This paper provides unique insight into selected food-literacy behaviours, food literacy related practices, dietary behaviours and the demographic characteristics of participants on enrolment in a food literacy program. This is the first time that factors from a reliability analysis have been used to explore different domains of food literacy. In addition, multivariate analysis has identified a subgroup of participants who will benefit most from a food literacy program. Although government targets indicate that low income and disadvantaged groups are often the focus for these programs [49,50], among the participants in this study, socio-economic disadvantage was not independently associated with the lowest food-literacy behaviours. These findings suggest that food literacy programs must focus on recruiting those with low self-rated cooking skills, who consider healthy foods expensive and have poor dietary intakes (i.e., are less likely to eat recommended serves of fruits and vegetables and have high takeaway and sugary drink consumption).

There was not strong evidence for segmenting programs for specific demographic subgroups, such as younger adults and males, as has been found by other researchers using nationally representative populations [4,18]. In part, this may be the result of many of the program evaluations (including this one) having mostly female participants from lower income groups [26,29]. In this study, the gender of participants was not independently associated with food literacy behaviours. In other analyses, its association was reported as mixed. There are associations between lower cooking confidence and being male and having a low income [23]; others studies have not found significant differences between levels of cooking confidence for gender, age or presence of children [22,51]. However, low confidence was identified in those who were less educated [22]. It was apparent that lower education levels and younger age groups were associated with the plan and manage domain of food literacy, which has also been found in Ireland [4]. The plan and manage domain of food literacy may be more useful in food literacy programs for certain subgroups—such as younger adults—for whom these may be new skills. A higher likelihood of individuals with low selection food literacy born outside Australia was discovered. This may be related to unfamiliarity with supermarkets and the types of foods available, unfamiliarity with nutrition information panels and food label formats or factors such as Halal dietary requirements [52].

It is common that females are over-represented in these programs, as they might be targeted as the person with the main responsibility for planning, selection and preparation in their household. The people with the main responsibilities have been referred to as the gatekeepers of household nutrition [53] and have traditionally been female. However, according to the Time Use Survey data, in high income countries, the time that men and women spend preparing food has been changing [17,54,55]. Participants in this food literacy program could be considered gatekeepers, as they were more likely to have the sole responsibility for meals and shopping and the age distribution was skewed towards middle or older adults [29]. Secondary analysis of the US National Health and Nutrition Examination Survey showed that most women and men reported sharing meal planning, preparing and food shopping activities (meal planning and preparation: women 54% and men 56%, food shopping: women 60% and men 57%), but women had a higher likelihood of reporting having the main responsibility for meal planning and food shopping [41]. Research with well-educated dietary gatekeepers in Australia who were recruited through market research demonstrated the role of food literacy for overcoming barriers to healthy eating and positive intentions for healthy food preparation [56]. Efforts to improve food literacy in dietary gatekeepers will have positive effects on diet quality for other household members [57].

The results demonstrate the importance of considering the participant’s cooking skills and indicate an independent association with dietary behaviours. This finding is similar to other studies, particularly when self-perceived cooking skills predict better dietary behaviours a decade later [58]. Burton et al. discovered that gatekeepers with high cooking confidence were significantly more likely to report using vegetables and meal planning [22]. McGowan et al. found that greater cooking skills were the strongest contributor in regression analyses for lower fat intakes, in addition to predicting higher fibre intakes and Eating Choices Index scores. However, they also discovered a myriad of other socio-demographic factors implicated in healthier dietary choices [4]. High levels of cooking self-efficacy and skills and an increased frequency of applying these skills have been proved to affect diet quality and be related to food security. Higher levels are related to increased fruit and vegetable, grain and protein consumption and decreased consumption of convenience foods and sugar-sweetened beverages [59,60].

Food literacy programs that aim to build and reinforce cooking confidence will be more likely to observe positive dietary and food literacy behaviour outcomes [25,28,29]. Although cooking skills are an important predictor of food literacy behaviours, they should not be an intervention target on their own [4]. Many factors influence food choices [61]; for example, in this research, a negative attitude to the cost of healthy foods was independently associated with low food literacy. If the perceived cost of healthy foods is high, participants may be less likely or unable to purchase and consume these foods [62]. As demonstrated in these results, interventions must focus on all domains of food literacy, to build what has been recently referred to as ‘food literacy proficiency’ [13].

Food literacy skills are developed over a person’s life and must be adapted to changing circumstances, such as moving out of home, changing household size (e.g., the birth of children), economic circumstances (e.g., changing income levels) and lifestyle factors (e.g., diagnosis of a lifestyle-related disease such as diabetes or high blood pressure). People with high food literacy attend food literacy programs [63], which suggests that programs operate more subtly to reinforce or remind participants to use their food literacy skills. The reasons that participants gave for attending FSA demonstrated that learning new ideas about cooking was the second most popular response. Programs might be used by some to ‘check in’ on their skills, to reinforce that they are doing well or to motivate small changes. Department of Health WA monitoring data from the 2015 NMSS discovered that most respondents reported knowing more about quicker ways to prepare healthy foods (82.1%), knowing of more ways to prepare healthier foods (75%) and knowing more about cooking (61.2%) would help them and their families to eat a healthier diet [40]. FSA enables all participants, even those with high food literacy, to safely and without cost try new ingredients and meals in a social environment.

Several limitations need to be considered in assessing the generalisability of these results. This is a cross-sectional study of the sample enrolled in the adult food-literacy program and participants were not randomly selected. Not all participants completed the evaluation, which might represent self-selection bias. It is likely that participants who did not complete the evaluation were from culturally and linguistically diverse groups who were possibly the least food literate in an Australian context. The validation of questions for assessing food literacy related behaviours limited the number of questions included in the evaluation tool due to considerations of respondent burden, cognitive load and reading level. There may be other variables associated with low food literacy that were not tested. The data was self-reported and may be subject to a social desirability bias [35]. Self-reported cooking skills might reflect self-confidence regarding the ability to cook, rather than actual capabilities and frequency of using these skills [22]. Further, confident cooks may not always be healthy cooks.

## 5. Conclusions

Food literacy programs are designed to help develop the knowledge, attitudes and skills required to make healthy food choices. Understanding the characteristics of participants in food literacy programs—and who benefits the most—enables program facilitators to target programs to specific groups and design curricula to achieve confidence and skills in key areas. Where the aim is to target those with low food literacy, identifying and marketing programs to those with low self-rated cooking skills and poorer dietary intake is critical.

## Figures and Tables

**Table 1:** Demographic characteristics of FSA participants 2016-2018.

Characteristic	Responses	FSA (%)
**Sex** (n = 1623)	Female	80.2
	Male	19.8
**Age** (n = 1626)	18–25y	13.5
	26–35y	23.9
	36–45y	23.3
	46–55y	13.5
	56–65y	12.7
	66 and over	13.2
**Household Composition**	Couple with children	35.7
(n = 1617)	Single person	16.3
	Partner	17.6
	Single parent with child/children	10.0
	Family/Extended family	10.0
	Shared/ Supported accommodation	10.4
**Education level**	Certificate/Diploma/Trade	33.3
(n = 1609)	Finished high school	23.6
	Bachelor or higher	24.2
	Some secondary/ finished primary	18.8
	Unemployed	25.8
**Employment status**	House duties/unable to work/retired	40.2
(n = 1607)	Part-time/ casual	22.8
	Full-time	11.2
Socioeconomic Index *	Low	43.8
(n = 1558)	Middle	29.5
	High	26.6
**Born in Australia ^†^** (n = 1508)		58.1
**Identify as Aboriginal or Torres Strait Islander ^†^** (n = 1496)		7.3

* SEIFA derived from postcode (Index of Relative Socio-economic Disadvantage) [43]. **^†^** Added in a later version of the questionnaire.

**Table 2:** Food literacy related practices and dietary behaviours compared to Western Australian Department of Health’s Nutrition Monitoring and Surveillance Survey 2015.

Food Literacy Related Practices and Dietary Behaviours	FSA % (n)	NMSS 2015 % (n)
**Responsibility for choosing and preparing meals in household**		
Sole responsibility	58.7	39.1
Shared responsibility	32.2.	51.5
No responsibility	7.0	9.3
	(n = 1604)	(n = 1205)
**Responsibility for household shopping**		
Sole responsibility	55.8	41.0
Shared responsibility	36.3	42.6
No responsibility	8.0	16.4
	(n = 1600)	(n = 1205)
**Self-rated cooking skills**		
Can cook almost anything	25.2	17.0
Can cook a wide variety	42.8	42.6
Can cook basic meat and 3 veg meal	23.6	17.8
Can boil an egg, BBQ meat or heat frozen meals	5.5	1.7
Can’t cook/don’t cook	2.9	1.0
	(n = 1605)	(n = 1205)
Fruit *	42.5	62.0
(≥2 recommended serves)	(n = 1459)	(n = 1205)
Vegetables *	5.6	12.2
(≥5 recommended serves)	(n = 1450)	(n = 1141)
Sugar sweetened drinks *		
Never consume	47.9	-
Try to always avoid	-	52.1
	(n = 1461)	(n = 1206)
Takeaway food consumption *		
Never consume	28.6	-
Don’t buy takeaway meals	-	2.7
	(n = 1460)	(n = 1206)

* Added in version 2 of the pre-program questionnaire.

**Table 3:** Summary of variables predicting low food literacy behaviours.

Predictor Variables *	Low Plan & Manage Behaviour Group	Low Selection Behaviour Group	Low Preparation Behaviour Group	Low Composite Food Literacy Behaviour Group †
Age	✔			
Education	✔			
Born outside Australia		✔		
Attitude to healthy food costs		✔	✔	✔
Responsibility for shopping	✔			
Responsibility for meals			✔	
Self-rated cooking skills	✔	✔	✔	✔
Serves of fruit	✔	✔	✔	✔
Serves of vegetables	✔	✔	✔	✔
Takeaway food frequency	✔		✔	✔
Sugar sweetened drinks frequency		✔		✔

* Other variables were non-significant of all 3 factor variables. **†** Identified as low on at least one factor.

**Table 4:** Demographic, dietary intake and food-related skill variables and their association with low food literacy behaviours. Numerical values denote the odds ratio with 95% confidence interval in brackets followed by the p value.

Demographic and Dietary Behaviours	Plan & Manage (n = 1249)		Selection (n = 1283)		Preparation (n = 1271)		Composite Identified as Low on at least 1 Factor	
	Univariable	Multivariable	Univariable	Multivariable	Univariable	Multivariable	Univariable	Multivariable
**Sex**								
Male	1		1		1		1	
Female	2 (1.54–2.59) *p* < 0.0001		1.21 (0.92–1.59) *p* = 0.1674		1.98 (1.53–2.56) *p* < 0.0001		1.57 (1.22–2.01) *p* = 0.0004	
**Age**								
18–25	1	1	1		1		1	
26–35	3.8 (2.45–5.91) *p* < 0.0001	2.57 (1.4–4.7) *p* = 0.0023	1.71 (1.12–2.62) *p* = 0.0127		2.07 (1.37–3.14) *p* = 0.0006		2.98 (2.01–4.41) *p* < 0.0001	
36–45	1.84 (1.23–2.76) *p* = 0.0032	2.12 (1.22–3.69) *p* = 0.008	1.21 (0.82–1.79) *p* = 0.3344		1.2 (0.82–1.76) *p* = 0.349		1.52 (1.08–2.13) *p* = 0.0153	
46–55	1.5 (1–2.27) *p* = 0.0527	1.44 (0.82–2.54) *p*=0.2023	0.97 (0.65–1.45) *p* = 0.8879		1.29 (0.88–1.89) *p* = 0.1884		1.46 (1.04–2.06) *p* = 0.0275	
56–65	1.88 (1.2–2.95) *p*=0.0058	2.18 (1.2–3.97) *p* = 0.0106	1.23 (0.8–1.9) *p* = 0.3494		1.12 (0.73–1.71) *p* = 0.6188		1.57 (1.07–2.3) *p* = 0.0197	
66 and over	1.55 (0.98–2.45) *p* = 0.0612	1.47 (0.8–2.73) *p* = 0.2158	0.82 (0.52–1.3) *p* = 0.3943		1.28 (0.84–1.97) *p* = 0.2549		1.49 (1.01–2.19) *p* = 0.0437	
**Education**								
Some high school	1	1	1		1		1	
Completed high school	3.2 (2.28–4.49) *p* < 0.0001	2.7 (1.71–4.25) *p* < 0.0001	2.22 (1.57–3.13) *p* < 0.0001		1.64 (1.19–2.27) *p* = 0.0026		2.11 (1.55–2.87) *p* < 0.0001	
Completed TAFE/Certificate/Diploma/Trade	1.98 (1.43–2.74) *p* < 0.0001	1.84 (1.19–2.83) *p* = 0.0059	1.73 (1.23–2.41) *p* = 0.0014		1.36 (1–1.85) *p* = 0.0506		1.7 (1.28–2.26) *p* = 0.0003	
Completed university degree (undergraduate or higher)	1.23 (0.9–1.68) *p* = 0.1946	1.35 (0.91–2.02) *p* = 0.1349	1.35 (0.98–1.86) *p* = 0.0624		0.9 (0.67–1.2) *p* = 0.4724		1.2 (0.92–1.56) *p* = 0.1777	
**Household Composition**								
Single person	1		1		1		1	
Couple with no children	0.55 (0.37–0.81) *p* = 0.0028		0.64 (0.44–0.94) *p* = 0.0223		0.56 (0.39–0.82) *p* = 0.0026		0.64 (0.46–0.89) *p* = 0.009	
Single parent with children	1.27 (0.83–1.92) *p* = 0.2704		1.29 (0.85–1.96) *p* = 0.2256		0.92 (0.61–1.39) *p* = 0.6894		1.35 (0.91–2.01) *p* = 0.1392	
Couple with children	0.76 (0.55–1.06) *p* = 0.1061		0.64 (0.46–0.89) *p* = 0.0074		0.72 (0.53–0.99) *p* = 0.0442		0.77 (0.57–1.03) *p* = 0.0738	
Other (e.g. shared or supported accommodation, family or extended family)	1.65 (1.16–2.34) *p* = 0.0053		0.95 (0.67–1.35) *p* = 0.7683		1.08 (0.77–1.53) *p* = 0.6453		1.23 (0.88–1.7) *p* = 0.2221	
**Employment**								
Full time/self employed	1		1		1		1	
Part time/casual	1.12 (0.77–1.63) *p* = 0.5498		0.82 (0.55–1.21) *p* = 0.3199		0.98 (0.68–1.4) *p* = 0.8945		0.92 (0.66–1.29) *p* = 0.64	
Unemployed/Unable to work/ disability pension/rehabilitation/ prison	1.36 (1.02–1.82) *p* = 0.0353		0.98 (0.73–1.31) *p* = 0.8674		1 (0.76–1.33) *p* = 0.9807		1.16 (0.9–1.5) *p* = 0.2572	
Other: Student/maternity leave/ retired/house duties/volunteer	2.17 (1.65–2.84) *p* < 0.0001		1.4 (1.06–1.84) *p* = 0.0164		1.55 (1.19–2.02) *p* = 0.0013		1.58 (1.24–2.03) *p* = 0.0003	
**SEIFA ^1^**								
High	1		1		1		1	
Middle	1.12 (0.85–1.48) *p* = 0.4119		1.11 (0.84–1.46) *p* = 0.4783		0.93 (0.71–1.2) *p* = 0.5742		1.1 (0.86–1.4) *p* = 0.4606	
Low	1.13 (0.84–1.52) *p* = 0.4227		1 (0.74–1.36) *p* = 0.9991		0.65 (0.49–0.88) *p* = 0.0044		1.01 (0.78–1.32) *p* = 0.926	
**Born in Australia ^2^**								
Yes	1		1	1	1		1	
No	1.23 (0.98-1.55) *p* = 0.0702		1.41 (1.11–1.78) *p* = 0.0043	1.38 (1.05–1.81) *p* = 0.0215	1.07 (0.86–1.34) *p* = 0.5448		1.2 (0.97–1.47) *p* = 0.0865	
**Identify as ATSI ^2,3^**								
Yes	1		1		1		1	
No	1.26 (0.83–1.91) *p* = 0.2783		1.73 (1.15–2.6) *p* = 0.008		0.7 (0.45–1.09) *p* = 0.1132		1.33 (0.9–1.98) *p* = 0.1526	
**Responsibility for shopping**								
All	1	1	1		1		1	
Some	0.98 (0.78–1.24) *p* = 0.8816	0.86 (0.63–1.17) *p* = 0.3268	0.85 (0.67–1.08) *p* = 0.1861		0.98 (0.78–1.23) *p* = 0.8777		0.95 (0.77–1.17) *p* = 0.6254	
None	4.29 (2.84–6.49) *p* < 0.0001	2.57 (1.42–4.65) *p* = 0.0018	1.58 (1.07–2.35) *p* = 0.0228		2.46 (1.67–3.64) *p* < 0.0001		2.48 (1.65–3.72) *p* < 0.0001	
**Responsibility for choosing and preparing the household meals**								
All	1		1		1	1	1	
Some	1.15 (0.91–1.46) *p* = 0.2353		0.94 (0.74–1.2) *p* = 0.6275		1.17 (0.93–1.47) *p* = 0.1879	0.97 (0.72–1.3) *p* = 0.8244	1.16 (0.94–1.44) *p* = 0.1611	
None	5.82 (3.69–9.17) *p* < 0.0001		2.49 (1.66–3.74) *p* < 0.0001		4.6 (2.99–7.07) *p* < 0.0001	2.43 (1.32–4.46) *p* = 0.0042	3.74 (2.35–5.93) *p* < 0.0001	
**Self-described cooking skills**								
Can cook almost anything	1	1	1	1	1	1	1	1
Can cook a wide variety of meals	1.22 (0.9–1.67) *p* = 0.2044	1.3 (0.88–1.92) *p* = 0.1938	0.91 (0.68–1.21) *p* = 0.5091	0.93 (0.67–1.29) *p* = 0.6534	1.72 (1.23–2.4) *p* = 0.0016	1.81 (1.22–2.68) *p* = 0.0033	1.42 (1.1–1.83) *p* = 0.0064	1.46 (1.08–1.96) *p* = 0.0143
Can cool a basic meat and 3 vegetables	3.94 (2.84–5.48) *p* < 0.0001	3.89 (2.56–5.92) *p* < 0.0001	1.51 (1.1–2.07) *p* = 0.01	1.19 (0.82–1.71) *p* = 0.3634	8.46 (5.95–12.04) *p* < 0.0001	8.67 (5.69–13.22) *p* < 0.0001	4.05 (3–5.46) *p* < 0.0001	3.61 (2.52–5.18) *p* < 0.0001
Can do basic heating of food, use barbeque, boil egg	11.39 (6.53–19.86) *p* < 0.0001	6.46 (3.2–13.03) *p* < 0.0001	2.81 (1.74–4.54) *p* < 0.0001	2.56 (1.44–4.55) *p* = 0.0014	20.28 (11.31–36.36) *p* < 0.0001	12.91 (6.29–26.49) *p* < 0.0001	9.37 (5.11–17.16) *p* < 0.0001	7.63 (3.61–16.12) *p* < 0.0001
Can’t cook/Don’t cook	15.25 (7.03–33.09) *p* < 0.0001	8.34 (2.85–24.35) *p* = 0.0001	2.81 (1.48–5.32) *p* = 0.0016	1.67 (0.73–3.82) *p* = 0.2219	22.06 (10.04–48.48) *p* < 0.0001	14.66 (4.92–43.7) *p* < 0.0001	7.38 (3.47–15.69) *p* < 0.0001	4.29 (1.51–12.14) *p* = 0.0061
**Healthy foods cost more**								
Disagree	1		1	1	1	1	1	1
Not sure	2.24 (1.64–3.04) *p*<0.0001		2.43 (1.78–3.32) *p* < 0.0001	1.97 (1.37–2.84) *p* =0.0003	2.97 (2.19–4.02) *p* < 0.0001	2.38 (1.61–3.53) *p* < 0.0001	2.3 (1.73–3.04) *p* < 0.0001	1.61 (1.14–2.28) *p* = 0.0074
Agree	1.89 (1.46–2.45) *p* < 0.0001		1.74 (1.33–2.28) *p* < 0.0001	1.6 (1.17–2.18) *p* = 0.003	1.99 (1.54–2.57) *p* < 0.0001	1.66 (1.19–2.3) *p* = 0.0027	1.87 (1.49–2.34) *p* < 0.0001	1.53 (1.16–2.02) *p* = 0.0029
**How many times a week on average do you eat fast food meals, such as burgers, pizza, chicken or chips from fast food outlets?**								
	1	1	1		1	1	1	1
Less than once a week	1.71 (1.25–2.33) *p* = 0.0009	1.28 (0.87–1.88) *p* = 0.2054	0.95 (0.71–1.27) *p* = 0.7304		1.49 (1.11–2) *p* = 0.0079	1.18 (0.82–1.69) *p* = 0.3637	1.41 (1.09–1.82) *p* = 0.0081	1.08 (0.8–1.47) *p* = 0.6237
Once or twice a week	3.66 (2.64–5.08) *p* < 0.0001	2.43 (1.62–3.66) *p* < 0.0001	1.43 (1.05–1.94) *p* = 0.0222		2.55 (1.87–3.47) *p* < 0.0001	1.62 (1.11–2.38) *p* = 0.0132	2.99 (2.24–3.99) *p* < 0.0001	1.73 (1.21–2.47) *p* = 0.0028
Three or more times a week	10.09 (5.78-17.63) *p* < 0.0001	5.7 (2.86–11.35) *p* < 0.0001	2.02 (1.22–3.34) *p* = 0.0061		5.67 (3.37–9.53) *p* < 0.0001	4.45 (2.33–8.52) *p* < 0.0001	5.65 (3.15–10.13) *p* < 0.0001	2.9 (1.43–5.91) *p* = 0.0033
**How many times a week on average do you drink regular soft drink (not diet), energy drinks, sports drinks, flavoured mineral water or vitamin water?**								
Never	1		1	1	1		1	1
Less than once a week	1.97 (1.46–2.66) *p* < 0.0001		1.64 (1.22–2.21) *p* = 0.0012	1.46 (1.05–2.03) *p* = 0.0229	1.73 (1.3–2.31) *p* = 0.0002		1.7 (1.3–2.21) *p* = 0.0001	1.27 (0.93–1.74) *p* = 0.1387
Once or twice a week	2.7 (1.94–3.76) *p* < 0.0001		1.82 (1.3–2.55) *p* = 0.0005	1.44 (0.99–2.09) *p* = 0.0544	2.26 (1.64–3.12) *p* < 0.0001		2.78 (2.03–3.83) *p* < 0.0001	1.85 (1.26–2.73) *p* = 0.0017
Three or more times a week	4.02 (2.88–5.6) *p* < 0.0001		2.95 (2.12–4.09) *p* < 0.0001	2.06 (1.42–2.99) *p* = 0.0001	2.47 (1.79–3.42) *p* < 0.0001		3.26 (2.33–4.54) *p* < 0.0001	1.94 (1.29–2.93) *p* = 0.0016
**Self-reported fruit intake**								
	0.54 (0.48–0.62) *p* < 0.0001	0.68 (0.57–0.8) *p* < 0.0001	0.68 (0.6–0.78) *p* < 0.0001	0.82 (0.71–0.95) *p* = 0.0094	0.67 (0.59–0.75) *p* < 0.0001	0.83 (0.71–0.97) *p* = 0.0187	0.66 (0.59–0.73) *p* < 0.0001	0.81 (0.71–0.92) *p* = 0.0017
**Self-reported vegetable intake**								
	0.53 (0.48–0.6) *p* < 0.0001	0.64 (0.56–0.74) *p* < 0.0001	0.71 (0.64–0.78) *p* < 0.0001	0.81 (0.72–0.91) *p* = 0.0006	0.61 (0.55–0.68) *p* < 0.0001	0.72 (0.63–0.83) *p* < 0.0001	0.63 (0.58–0.69) *p* < 0.0001	0.75 (0.67–0.83) *p* < 0.0001
